# Identifying extended psychosis phenotypes at school: Associations with socio-emotional adjustment, academic, and neurocognitive outcomes

**DOI:** 10.1371/journal.pone.0237968

**Published:** 2020-08-21

**Authors:** Beatriz Lucas-Molina, Alicia Pérez-Albéniz, Encar Satorres, Javier Ortuño-Sierra, Elena Domínguez Garrido, Eduardo Fonseca-Pedrero

**Affiliations:** 1 Department of Developmental and Educational Psychology, University of Valencia, Valencia, Spain; 2 Department of Educational Sciences, University of La Rioja, Logroño, Spain; 3 Programa Riojano de Investigación en Salud Mental (PRISMA), University of La Rioja, Logroño, Spain; 4 Molecular Diagnostic Unit, Fundación Rioja Salud, Logroño, La Rioja, Spain; 5 Centro de Investigación Biomédica en Red de Salud Mental (CIBERSAM), Department of Psychiatry, University of Oviedo, Oviedo, Spain; University of Sao Paulo Medical School, BRAZIL

## Abstract

The main goal of the present study was to explore the latent structure of extended psychosis phenotypes in a representative sample of adolescents. Moreover, associations with socio-emotional adjustment, academic achievement, and neurocognition performance across the latent profiles were compared. Participants were 1506 students, 667 males (44.3%), derived from random cluster sampling. Various tools were used to measure psychosis risk, subjective well-being, academic performance, and neurocognition. Based on three psychometric indicators of psychosis risk (schizotypal traits, psychotic-like experiences, and bipolar-like experiences), four latent classes were found: non-risk, low-risk, high reality distortion experiences, and high psychosis liability. The high-risk latent groups scored significantly higher on mental health difficulties, and negative affect, and lower on positive affect and well-being, compared to the two non-risk groups. Moreover, these high-risk groups had a significantly higher number of failed academic subjects compared to the non-risk groups. In addition, no statistically significant differences in efficiency performance were found in the neurocognitive domains across the four latent profiles. This study allows us to improve the early identification of adolescents at risk of serious mental disorder in school settings in order to prevent the incidence and burden associated with these kinds of mental health problems.

## Introduction

Previous research has demonstrated that the reliable identification of individuals at high risk for psychotic-spectrum disorders and timely prophylactic intervention may delay, ameliorate, or prevent the onset of frank psychotic symptoms [1,2]. Prevention of serious mental disorders may reduce their possible negative effects at different levels (e.g., personal, familiar, academic, work, health, economic) [3]. Thus, it is crucial to provide new knowledge to accurately detect individuals who are at risk for the development of psychotic disorders, prior to clinical onset, in order to activate suitable prevention strategies [3–5]. The ideal window of opportunity to improve the outcomes of serious mental disorders is promotion of emotional well-being or prevention in children and adolescents at both school and clinical settings [6,7].

Schools are a logical context in which prevention strategies of mental disorders in youth populations have to be implemented. On the one hand, the vast majority of children and adolescents attend school, and they do it at an age prior to the development of the first symptoms of psychosis [8] as well as other mental disorders [9]. In fact, onset of psychological disorders during adolescence has been related with underperformance in school [10]. On the other hand, according to the National Association of School Psychologists (NASP), psychologists are expected to develop, implement, and evaluate prevention and intervention programs based on risk and protective factors that are precursors to severe mental health problems [11]. Indeed, schools are the most common entry point for youth to access mental and behavioral health services [12]. Despite this fact, only half of the children in need of mental health services actually receive help [13]. Hence, schools have a key role in the early identification and prevention of individuals at risk for mental disorders (e.g., psychosis) as well as in promoting the optimal child and adolescent development. However, to achieve this goal adequate measurement tools [14,15] and empirically validated treatments are needed [16,17].

One of the well-known liability marker for psychosis spectrum disorders is called schizotypy [18], which can be measured by genetic, psychometric, laboratory, or/and clinical indicators [19]. Prior research has shown that schizotypal traits (e.g., anhedonia, distortion reality, interpersonal) and psychotic-like experiences may represent the behavioral manifestation of a distributed multifactorial risk of psychosis [5,20–24]. For instance, Radua et al. [21], conducting an umbrella review, found that the subclinical expression of psychosis phenotype as ultra-high-risk state for psychosis and trait anhedonia were the main risk factors for psychosis with convincing or highly suggestive evidence of association. In addition, previous follow-up studies have showed that adolescents and young adults who report subclinical expressions of psychosis phenotype (e.g., Psychotic Like Experiences-PLEs-, schizotypal traits) have a greater probability of clinical outcome, mainly non-affective psychoses; however; the positive predictive values estimated are too low [5,16,20–21]. Thus, in order to improve our prevention capacity, we need to incorporate known risk factors within (e.g., schizotypy, PLEs) and between multiple levels of analysis (e.g., genetic, psychophysiological, cognitive, psychopathological, demographic). In addition, adolescents with psychosis liability (e.g., higher scores on schizotypy/schizotypal measures) reported a higher prevalence of mental disorders, as well as psychopathology symptoms related to depression, anxiety, and suicidal behaviors, among others [25–30].

There is an increasing interest in finding homogenous groups of potentially at-risk individuals based on psychotic-spectrum phenomena [31,32]. A novel mixture model called latent class analysis (LCA) (dichotomous outcome) or latent profile analysis (LPA) (continuous outcome) [33] was recently used for this purpose. In this regard, previous studies have examined the latent structure across the psychosis phenotype in both clinical and school settings [29,34–37]. For instance, Cella et al. [29] used LCA in a large sample of non-clinical adolescents. They found three classes: low schizotypy, unusual subjective experiences, and true schizotypy. The adolescents in the true schizotypy latent class reported more psychological distress and family history of psychosis, compared to the other classes. In other study, Fonseca-Pedrero et al. [31], using a representative sample of adolescents, found four latent profiles (positive schizotypy, low schizotypy, social disorganization schizotypy, and high schizotypy), where the high schizotypy class scored higher on psychopathology indicators relative to the other three clusters.

To date, however very little is known about the latent structure of psychosis extended phenotypes during adolescence. For instance, previous studies have only used one psychometric index of psychosis risk (e.g., schizotypy measure) to latent class identification. Likewise, there has been no in-depth examination of the relationship between psychosis liability latent profiles and their link to socio-emotional, academic, and neurocognitive variables. Therefore, new studies are needed to identify subclinical psychosis phenotypes at risk, integrating different but complementary detection approaches (e.g., combining schizotypal traits, prodromal psychotic symptoms, and bipolar-like experiences) and to validate them across multiple psychometric indicators, both subjective and objective, beyond traditional paper-pencil assessment. Thus, this kind of studies may enhance the accuracy and validity of early identification and detection before clinical transition during a sensitive developmental period at school-based settings.

Within this framework, the main goal of the present study was to explore the latent structure of multidimensional extended psychosis phenotype in a large sample of non-clinical adolescents. Moreover, associations with socio-emotional adjustment (emotional and behavioral problems, negative and positive affect, and subjective wellbeing), academic performance, and neurocognition domains (executive function, episodic memory, complex cognition, and social cognition) across the latent profiles were examined in order to validate the theoretical subgroups found.

## Materials and methods

### Participants

Participants were 1506 students, 667 males (44.3%), from 34 schools and 98 classrooms. The sample was selected using stratified random cluster sampling, with the classroom as the sampling unit, from a population of 15,000 students in the region of La Rioja (northern Spain). The students belonged to different public and charter Secondary and Vocational Training Schools, and to different socio-economic levels. The layers were created as a function of the geographical zone and the educational stage.

The mean age was 16.5 years (*SD* = 1.36), with an age range between 14 to 19 years old. Distribution by age was: 14-year-olds (*n* = 200; 13.3%), 15-year-olds (*n* = 313; 20.8%), 16-year-olds (*n* = 381; 25.3%), 17-year-olds (*n* = 365; 24.2%), 18-year-olds (*n* = 174; 11.6%), and 19-year-olds (*n* = 73; 4.8%).

In the present study, the inclusion criteria were: a) to provide written informed consent; b) to take all psychometric tests and neurocognitive battery, in that regard, 1141 participants fully completed the neurocognitive battery; and c) an age between 14 and 18 years. The exclusion criteria were as follows: a) to score more than 3 points on the Oviedo scale of infrequency of response [38]; b) to have any mental disorders diagnosed by the school psychologist; and c) to have any medical or neurological illness.

### Instruments

#### The Oviedo Schizotypy Assessment Questionnaire-Revisited (ESQUIZO-Qr) [39]

This self-report assesses schizotypal traits in adolescents. It includes a total of 62 items rated on a 5-point Likert-type response scale (from 1 “*strongly disagree*” to 5 “*strongly agree*”) and grouped in 10 subscales, which, in turn, form three general dimensions: Reality Distortion, Anhedonia, and Social Disorganization. Previous studies have demonstrated that ESQUIZO-Qr showed adequate psychometric properties (internal consistency levels for the subscales range from 0.62 to 0.90) [37,39].

#### The Prodromal Questionnaire–Brief (PQ-B) [40]

The PQ-B is a psychosis-risk screening measure. It consists of 21 dichotomous items (true/false) and two additional Likert scale questions inquiring about frequency and related distress or impairment. The Spanish adaptation of the PQ-B has demonstrated adequate psychometric properties (Omega total score = 0.92 and unidimensional factor structure) [41].

#### The Mood Disorder Questionnaire (MDQ) [42]

It consists of 13 dichotomous yes/no items based on the DSM-IV criteria for bipolar disorder. If the participant endorses 7 or more of the 13 items, confirms that 2 or more symptoms occurred at the same time (Criterion 2), and rates the functional impairment as moderate to severe (Criterion 3), then the MDQ is considered positive. In this study, we used the Spanish version adapted and validated in adolescents and young adults (ordinal alpha for Energy-Activity factor = 0.94 ordinal alpha for Disinhibition-Attention factor = .89) [43].

#### The Strengths and Difficulties Questionnaire (SDQ) [44]

The SDQ is a self-report that has been widely used for the assessment of emotional and behavioural problems related to mental health in adolescents. It consists of 25 statements grouped in five subscales: Emotional symptoms, Conduct problems, Hyperactivity, Peer problems, and Prosocial behavior. The first four subscales yield a Total difficulties score. Respondents used a 3-point Likert scale (0 = “Not true”, 1 = “Somewhat true”, 2 = “Certainly true”). The Spanish version of the SDQ-self-report version was used in the present study [45]. The level of internal consistency of the Total difficulties score was .84, ranging between .71 and .75 for the SDQ subscales.

#### The Personal Well-being Index–School Children (PWI-SC) [46]

It comprises eight items, with response options ranging from 0 (“*completely dissatisfied*”) to 10 (“*completely satisfied*”). The PWI-SC items assess subjective satisfaction with a specific area of life (e.g., standard of living, health, life achievements). The total scale score is the result of adding up the scores on these 7 items, ranging from 0 to 70 points. The validation of the PWI-SC into Spanish has shown adequate psychometric properties (Alpha total score = .85) [47].

#### The 10-item Positive and Negative Affect Schedule for Children (PANAS-C) [48]

It consists of 10 items and two factors designed to measure Positive Affect (PA, happy, lively, happy, energetic, and proud) and Negative Affect (NA, depressed, angry, fearful, scared, and sad). Children have to indicate the extent to which they have experienced each emotion in the past few weeks on a 5-point Likert-scale from 1 (“*very slightly or not at all*”) to 5 (“*extremely or very much*”). In the present study, the Spanish version of the PANAS-C has been used (alpha for NA = .85; alpha for PA = .81) [47].

#### Assessment of academic performance

The students’ academic achievement was obtained through the following question: "*Did you fail any subject in the previous academic evaluation*?" with a Yes or No answer. If the answer was affirmative, the student had to specify the number of failed subjects.

#### The Penn Computerized Neurocognitive Battery (CNB) for children [49–51]

The CNB is a single 1-hour computerized battery that combines tests from multiples batteries, which is one of its main strengths. The battery includes a training module and 14 computerized tests assessing five neurocognitive domains. Each domain consists of two or three tests: Executive Function (Abstraction and Mental-Flexibility, Attention and Working-Memory), Episodic Memory (Words, Faces and Figures), Complex Cognition (Verbal reasoning, Nonverbal-reasoning and Spatial Processing), Social Cognition (Emotion Identification, Emotion intensity Differentiation and Age Differentiation), and Sensorimotor speed (Motor and Sensorimotor). A detailed description of the CNB is reported elsewhere [51]. Each test provides measures of both accuracy (number of correct responses) and speed (median time for correct responses), except the Sensorimotor and Motor Speed tests. The efficiency scores are the primary outcome of the CNB for each domain. They are calculated by averaging the accuracy and speed scores on each test. Therefore, there is no efficiency score for the Sensorimotor domain. For the purposes of the present study, we used the efficiency scores. Previous studies have shown adequate psychometric properties of the CNB [49,51,52]. The Spanish version of the CNB was used [53].

#### The Family Affluence Scale-II (FAS-II) [54]

Socioeconomic status was measured using a four-item, child-appropriate measure of family wealth, with scores ranging from 0 to 9. Previous international studies have demonstrated its adequate psychometric properties [54].

### Procedure

The instruments and neurocognitive battery were administered collectively via personal computers in groups of 10 to 30 students, during a standard two-hour session and in a classroom especially prepared for this purpose. All participants must complete each questionnaire in order to assure its validity and allow for its correct administration (i.e. there can be no missing values). Participants were free to withdraw from the study at any time. No incentive was provided for their participation.

### Ethics statement

The research was approved by the Ethical Committee for Clinical Research of La Rioja (CEICLAR). For participants under the age of 18, parents were asked to provide written informed consent. The investigation must have been conducted according to the principles expressed in the Declaration of Helsinki.

### Data analyses

First, we calculated descriptive statistics for all the continuous measures. Second, Pearson correlations between the ESQUIZO-Qr, PQ-B, and MDQ were conducted.

Third, in order to test for the existence of discrete groups (classes) with similar psychometric profiles, we conducted a latent profile analysis (LPA) using the ESQUIZO-Qr dimensions, and PQ-B (frequency and distress), and MDQ total scores. To improve comparability, all of them were transformed into *z*-scores. In LPA, models are compared to determine the optimal number of classes (i.e., class enumeration), first evaluating the fit of a one-class model and incrementally adding latent classes until the best class solution has been satisfied. Model selection is based on the consideration of several fit indices: Akaike Information Criterion (AIC), the Bayesian Information Criterion (BIC); the sample-size adjusted BIC (ssaBIC); and Lo-Mendell-Rubin’s adjusted likelihood ratio test (LRT). The standardized measure of entropy was also computed in order to assess (ranges from 0 to 1) the relative accuracy of the participants’ classification, with higher values indicating better separation of the identified groups [55].

Fourth, after determining the best latent profile solution, the effect of latent profile membership on mental health adjustment (SDQ, PANAS-C), personal wellbeing, academic indicators, and neurocognitive domains was analyzed using multivariate analysis of covariance (MANCOVA). Gender, estimated IQ, and socio-economic status were used as covariates. The Penn Matrix Reasoning Test of the CNB Battery-Child (Complex Cognition domain) was used to IQ estimation. The estimated IQ was not included as a covariate for the analysis of the neurocognitive domains as it was part of the Complex Cognitive dimension. Partial eta squared (η^2^) was used as the effect size index.

SPSS 22.0 [56] and Mplus 7.4 [57] were used for data analyses.

## Results

### Descriptive statistics of measures

The descriptive statistics (means, standard deviations, asymmetry, and kurtosis) are reported in Table 1. Table 2 shows the Pearson correlations among the five variables to be included in the LPA. As the table reveals, all the pairs of correlations were positive and significant (*p*<0.05), with the exception of the one between the Anhedonia dimension (ESQUIZO-Qr) and the MDQ.

**Table 1:** Descriptive statistics for all variables (N = 1506).

	Mean	SD	Asymmetry	Kurtosis
*LPA variables*				
ESQUIZO-Qr Reality Distortion	6.74	2.90	1.16	1.13
ESQUIZO-Qr Anhedonia	8.29	3.32	1.26	1.80
ESQUIZO-Qr Social Disorganization	11.00	4.97	1.69	2.89
PQ-B Frequency	6.03	4.39	0.62	-0.28
PQ-B Distress	11.10	11.47	1.64	3.48
MDQ	5.03	2.83	0.14	-0.61
*Mental health and wellbeing indicators*				
SDQ Total Score	11.37	5.19	0.42	-0.07
PANAS-C Negative Affect	10.77	3.62	0.45	-0.47
PANAS-C Positive Affect	18.02	2.88	-1.93	3.60
PWI-SC	55.10	9.00	-1.05	1.50
*Academic performance*				
Number of Failed Subjects	1.78	1.97	1.12	0.59
*Efficiency in neurocognitive domains (N = 1141)*				
Executive Function	0.04	0.82	-0.20	0.78
Episodic Memory	0.04	1.08	-0.23	0.57
Complex Cognition	0.02	1.25	0.12	-0.23
Social Cognition	0.01	1.12	0.02	0.17
*Covariates*				
PMRT (Estimated IQ)	5.37	4.51	0.35	-0.96
FAS-II (Socioeconomic Status)	6.14	1.69	-0.27	-0.38

LPA = Latent Profile Analyses; ESQUIZO-Qr = The Oviedo Schizotypy Assessment Questionnaire-Revised; PQ-B = The Prodromal Questionnaire-Brief; MDQ = The Mood Disorder Questionnaire; SDQ = The Strengths and Difficulties Questionnaire; PANAS = The 10-item Positive and Negative Affect Schedule for Children, PWI-SC = The Personal Wellbeing Index–School Children; MDQ = The Mood Disorder Questionnaire; PMRT = The Penn Matrix Reasoning Test; FAS-II = The Family Affluence Scale-II.

**Table 2:** Pearson’s correlations between the measures included in the latent profile analyses (N = 1506).

ESQUIZO-Qr	PQ-B Frequency	PQ-B Distress	MDQ
Reality Distortion	.661**	.645**	.354**
Anhedonia	.105**	.137**	.006
Social Disorganization	.567**	.541**	.338**

**p < .01.

ESQUIZO-Qr = The Oviedo Schizotypy Assessment Questionnaire-Revised; PQ-B = The Prodromal Questionnaire-Brief; MDQ = The Mood Disorder Questionnaire.

### Latent profile analyses: Identification of extended psychosis phenotypes classes

We computed five latent profile solutions. Table 3 provides the goodness-of-fit indices for the competing latent profile models of schizotypal traits, psychotic-like experiences (frequency and distress), and bipolar-like experiences. As the table reveals, the four-class model was the best-fitting one. It showed a significant LMR-A *p* value and lower AIC, BIC, and ssaBIC values than the competing two- and three-class models. By contrast, the five-class model showed a nonsignificant LMR-A *p* value and the lowest entropy value.

**Table 3:** Goodness-of-fit statistics for the latent profile solutions.

Model	Log-likelihood	AIC	BIC	ssaBIC	Entropy	LMR-A	LMR-A *p*
1	-12817.34	25658.68	25722.48	25684.36	-	-	-
2	-11455.10	22948.20	23049.23	22988.87	0.901	2672.30	0.000
3	-10988.58	22029.17	22167.42	22084.82	0.867	915.17	0.007
4	-10731.26	21528.52	21703.99	21599.15	0.867	504.80	0.021
5	-10658.34	21396.68	21609.37	21482.30	0.861	143.04	0.284

AIC = Akaike information criterion; BIC = Bayesian information criterion; ssaBIC = sample-size adjusted BIC; LMR-A = Lo-Mendell-Rubin-adjusted likelihood ratio test.

**Fig 1:**
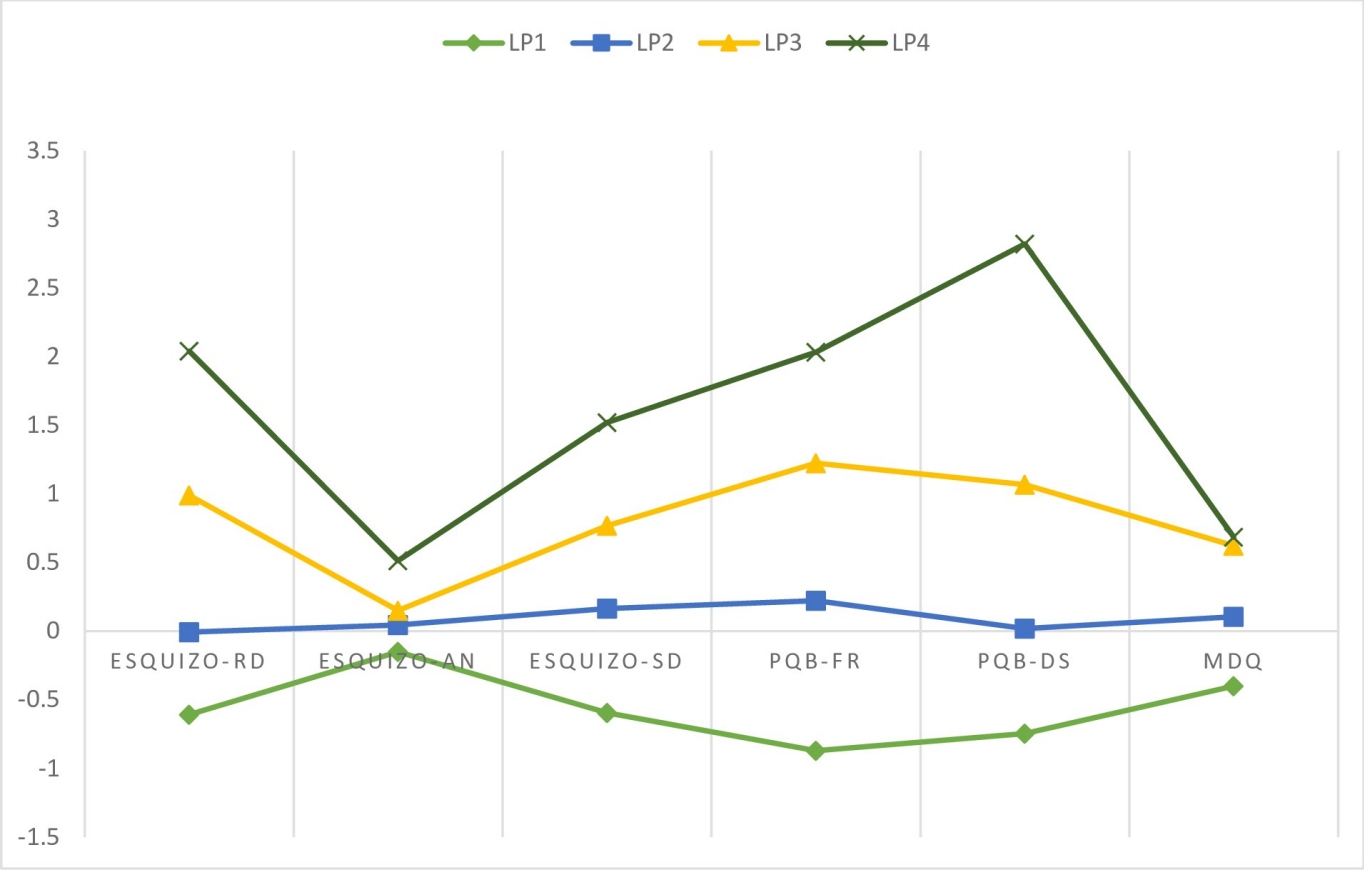
Latent Profile (LP) analysis: Four latent profile solution. ESQUIZO = The Oviedo Schizotypy Assessment Questionnaire-Revised; RD = Reality Distortion; AN = Anhedonia; SD = Social Disorganization; PQ-B = The Prodromal Questionnaire-Brief; PQB-FR = Frequency; PQB-DS = Distress; MDQ = The Mood Disorder Questionnaire.

In the four-class solution, latent profile 1 (LP1) described 44.2% (*n* = 665), LP2 17.1% (*n* = 257), LP3 33.8% (*n* = 509), and LP4 5% (*n* = 75) of the adolescents. The average class membership for LP1, LP2, LP3, and LP4 was 094, 0.92, 0.91, and 0.96, respectively, indicating good overall discrimination. Fig 1 illustrates this latent profile solution. LP1 members showed low scores on the schizotypal dimensions, psychotic-like experiences, and bipolar-like experiences. This group was called the “non-risk”. Participants in LP2 displayed medium scores (z close to zero) across all the schizotypy, psychotic, and bipolar facets, and they were called the “low-risk” group. Participants in LP3 displayed higher scores on all aspects, with the exception of the schizotypy dimension Anhedonia. We identified this class as “high reality distortion experiences”. LP4 members showed high scores on all the schizotypal, psychotic-like, and bipolar-like domains. This group was identified as having “high psychosis liability”.

No statistically significant differences were found by age (*F*_(3, 1587)_ = 2.070; *p* = 0.102), but there were significant differences by gender (χ^2^_(3)_ = 20.84; *p* < .001) across LP. There were more males in LP1 and more females in LP3 and LP4, compared to the other LPs.

### Latent profiles and socio-emotional adjustment

In order to examine the relationships between the four latent profiles and the five indicators of mental health and wellbeing (i.e., SDQ total score, PANAS-C, and PWI-SC), a MANCOVA was carried out. Gender, age, estimated IQ, and socioeconomic status were used as covariates. The MANCOVA revealed a significant overall main latent group effect [Wilk´s λ = 0.646, *F*_(12, 3955.69)_ = 59.097; *p* < 0.001; *partial η*^*2*^ = 0.135]. Moderate effect sizes were found (see Table 4). Bonferroni post-hoc analyses revealed that both of the high psychosis liability groups scored significantly higher on emotional and behavioral problems, and negative affect, and lower on positive affect and personal wellbeing, than the two non-risk groups.

**Table 4:** Mental health, wellbeing, academic performance and efficiency performance in neurocognitive domains across latent profiles.

		LC1 (n = 665)		LC2 (n = 257)		LC3 (n = 509)		LC4 (n = 75)					*Post hoc comparisons*
		*M*	*SD*	*M*	*SD*	*M*	*SD*	*M*	*SD*	*F*	*p*	*Partial η*^*2*^	
*Mental health and wellbeing indicators (N = 1506)*													
	SDQ total score	9.21	3.66	8.97	3.78	14.86	5.52	14.97	3.30	194.765	<0.001	0.281	1,2 < 3, 4
	PANAS-C Negative Affect	9.42	2.88	9.79	3.25	12.63	3.67	13.57	3.34	112.791	<0.001	0.184	1,2 < 3, 4
	PANAS-C Positive Affect	19.00	1.67	19.03	1.64	16.50	3.58	16.13	3.86	110.007	<0.001	0.181	1,2 > 3,4
	PWI-SC	58.10	6.83	57.78	5.83	50.46	10.56	50.73	8.62	95.892	<0.001	0.161	1,2 > 3,4
*Academic performance (N = 1506)*													
	Number of failed subjects	1.67	1.93	1.43	1.79	2.06	2.04	2.08	2.17	9.380	<0.001	0.018	3 > 1,2; 4 > 2
		LC1 (n = 429)		LC2 (n = 257)		LC3 (n = 380)		LC4 (n = 75)					*Post hoc comparisons*
		*M*	*SD*	*M*	*SD*	*M*	*SD*	*M*	*SD*	*F*	*p*	*Partial η*^*2*^	
*Efficiency in neurocognitive domains (N = 1141)*													
	Executive Control	0.03	0.86	0.10	0.78	0.02	0.79	0.52	0.82	0.551	.648	0.001	-
	Episodic Memory	0.04	1.06	0.22	0.99	0.12	1.02	-0.09	1.08	2.786	.040	0.007	-
	Complex Cognition	0.03	1.18	0.16	1.22	0.02	1.17	0.14	1.24	0.876	.453	0.002	-
	Social Cognition	0.03	1.08	0.08	0.98	-0.02	1.10	0.03	1.07	0.360	.782	0.001	-

LC = Latent class; M = Mean; SD = Standard deviation; SDQ = The Strengths and Difficulties Questionnaire; PANAS-C = The 10-item Positive and Negative Affect Schedule for Children; PWI-SC = The Personal Wellbeing Index–School Children.

### Latent profiles and academic performance

An indicator of academic performance was used: the number of failed subjects in the previous school year. Gender, age, estimated IQ and SES were included as covariates. The results of the ANCOVA revealed statistically significant differences [*F*_(3, 1498)_ = 9.380, *p* < 0.001, *Partial η*^*2*^ = 0.018] in the number of failed subjects across the latent profiles. As Table 4 reveals, Bonferroni post-hoc analyses showed that the LP3 group had a significantly higher number of failed subjects than the LP1 and LP2 groups. The LP4 group had significantly more failed subjects than the LP1 group. No differences were found between the two risk groups or between the two non-risk groups.

### Latent profiles and neurocognitive performance

The MANCOVA on efficiency performance in the different neurocognitive domains did not reveal a main effect of latent group [Wilk’s *λ* = 0.958, *F*_(12, 2992.636)_ = 1.158, *p =* 0.308, *partial η*^*2*^ = 0.004]. Gender, age, and SES were included as covariates. In this case, we did not include IQ because it was part of the Complex Cognitive dimension. Therefore, there were no statistically significant differences in efficiency performance in the neurocognitive domains across the four latent profiles (see Table 4).

## Discussion

The main goal of the present study was to identify homogeneous subgroups of adolescents based on multiple psychosis liability indicators, i.e., schizotypal traits, psychotic-like experiences, and bipolar-like experiences. In addition, links to socio-emotional adjustment, academic achievement, and neurocognitive performance were compared across latent profiles. The reliable identification of homogeneous subgroups of students at risk for psychosis spectrum disorder in school settings, may open up the possibility of developing prophylactic preventive interventions and understanding tentative etiological mechanisms as well as risk and protective factors.

First, the results showed that the best fitting latent profile model was obtained with a four-class solution, consisting of the following: “non-risk” (44.2%), “low-risk” (17.1%), “high reality distortion experiences” (33.8%), and “high psychosis liability” (5%). Therefore, two of the profiles (61.3%) could be identified as the “healthy” groups, whereas the other two (38.8%) could be considered to have a theoretical “high risk” of serious mental disorder. The prevalence of the high-risk groups is similar to what was reported in previous studies (around 10%) and taxometric analyses [18,22,58]. To date, few attempts have been made to identify latent classes of adolescents potentially at high risk of psychosis using multiple psychosis risk measures. In fact, integrating different but complementary detection approaches of psychosis risk may improve our predictive capacity. Taking this into account, these findings are consistent with the few prior studies of schizotypy or psychotic-like experiences that used a latent class or cluster approach in adolescent and adult populations [29,34–37,59,60]. In particular, most of these studies identified at least three latent classes, including one class of adolescents who displayed low levels of schizotypy and adaptive functioning, and at least two classes showing higher levels of schizotypal traits.

In this regard, the Cella et al. [29] “low schizotypy group” could correspond to our “low-risk” and “non-risk” groups, whereas their “unusual subjective experiences” might correspond to our “high reality distortion experiences”. This group is tentatively characterized as being at moderate risk of developing a psychotic disorder. Finally, individuals in our “high liability” group (LP4) might be classified in “true schizotypy” group. In the present study, this group displays positive schizotypal traits accompanied by disorganization schizotypal traits and psychotic distress. Nevertheless, our findings differed from the Cella et al. [29] study because we did not merely focus on a simple psychosis-proneness measure. Instead, we included measures of schizotypal traits, psychotic-like experiences (frequency and distress), and bipolar-like experiences. This combination of psychometric indicators may allow us to better identify youth at risk of both affective and non-affective psychosis and other forms of psychopathology (e.g., emotional and behavioral problems) in school settings.

Furthermore, we would like to highlight the role of PQ-B distress scale that clearly distinguish between the LP3 and the LP4. In particular, the “high liability group” (LP4) displays a markedly higher distress score than the other three groups. The distress associated to psychotic experiences is a landmark in order to diagnose at risk mental states and frank psychotic symptoms [1,61,62]. Thus, these students could be characterized as a target group to develop prevention intervention [63]. In overall terms, these results address the question of whether it is possible to identify a homogenous subgroup of vulnerability from the adolescent general population, as well as if those potentially at high risk for psychosis are a true psychosis liability group.

The second goal of this study was to expand the validity of these latent profiles by examining their associations with mental health and wellbeing, academic achievement, and neurocognitive performance. Both of the high-risk groups scored significantly higher on emotional and behavioral problems and negative affect, and lower on positive affect and subjective well-being (low quality of life), compared to the two non-risk groups. Moreover, these high-risk groups had a significantly higher number of failed subjects compared to the non-risk groups. Previous studies with adolescents and young adults from both clinical and non-clinical populations have shown similar results to those found in this study. For instance, adolescents who reported psychotic-like experiences indicated a wide range of mental health problems, such as depressive symptoms, emotional and behavioral problems [27,64]. These results have potential implications. First, adolescents at risk have other current psychiatric comorbidities, e.g., internalization problems, even without a diagnosed clinical disorder. Second, it is plausible that a large percentage of individuals with psychosis liability do not go on to develop major psychopathology, but they could report certain mental symptoms, as well as low quality of life and mental distress [1,2]. Third, high-risk groups could allow us to prevent not only psychotic disorders, but also other mental health difficulties or problems.

Unexpectedly, there were no differences across the latent profiles in efficiency performance in the different neurocognitive domains (i.e., executive functions, episodic memory, complex cognition, and social cognition). Neurocognitive deficits are an integral characteristic of the psychosis-spectrum phenotype and might precede the onset of psychotic episodes and predict the onset of the illness [65–67]. Nonetheless, when a high risk of psychosis has previously been assessed in youths from the general population, the findings have been mixed [68–72]. For instance, Gur et al. [69] found that individuals who endorsed psychotic symptoms were neurocognitively delayed, compared to those with non-psychiatric disorders, with these differences being more pronounced in the complex and social cognition domains. However, Siddi et al. [71] also found evidence of worse verbal and visual-spatial working memory functioning, but not other cognitive domains, in people with schizotypy or with schizotypal traits. In a meta-analysis of 33 studies examining neurocognition in at-risk college students (based on schizotypal profiles), Chun et al. [68] revealed between-group differences in the negligible effect-size range in most of the neurocognitive domains. It is important to note that most of these findings were obtained in non-clinical adult populations (e.g., mainly college samples). In fact, to date, no previous studies have examined LPA using multiple psychosis risk indicators and a full neurocognitive battery in a large sample of adolescents derived from the general population. Thus, there is inconclusive evidence about how neurocognitive deficits manifest across the schizophrenia-spectrum. The absence of statistically significant differences in neurocognitive functioning between latent classes could have multiple explanations: a) it is possible that the tools used (self-report), do not really capture the risk of psychosis or that the standardized neurocognitive battery is not sensitive enough to capture the differences between groups; b) this is a sample of the general adolescent population, so it is possible that many of the participants do not have true risk for psychosis, and none of them even end up developing psychosis; c) it is possible that the first neurocognitive deficits have not yet manifested themselves since they are non-clinical adolescents; and d) according to Chun et al., [68] although at-risk individuals report relatively neurocognitive deficits at a subjective level, it is possible that the neurocognitive functioning assessed behaviorally is largely intact. Further studies should explore the relationship between neuropsychological functioning and extended psychosis phenotype in new representative samples of adolescents.

Overall, our findings suggest that the two subgroups of participants at potentially high risk for psychosis also present higher emotional and behavioral symptoms, negative affect and academic failure. Consequently, they may require attention and could benefit from further comprehensive assessments and psychoeducation on mental health and wellness. Indeed, although the presence of subclinical psychotic experiences or traits during adolescence is not a necessary or sufficient condition for the later development of a mental health disorder, the members of the LP4 may be likely to be eligible for further clinical high-risk screening. In that regard, considering the school setting, psychologists should preferably refer these students (*n* = 75) to the closest community mental health services for further clinical screening and assessment, and, if necessary, provide them with the most appropriate intervention. Certainly, the best course of action would be to also monitor the liability group based on high scores on reality distortion dimension (those reporting higher unusual perceptual experiences) (LP3, *n* = 509) over time, to see if this set of experiences persist across time, and whether they are associate with cognitive decline, affective disturbances, academic failure, and social isolation [73]. For instance, school psychologists should work collaboratively with both the students’ mental health providers and teachers, in order to prevent the emergence of further symptomatology, as well as to adapt the school context to suit the students’ needs.

The results obtained in the present study must be interpreted with the following limitations in mind. First, adolescence is a developmental period in which the brain, cognition, and personality are still consolidating, and these bio-psychological changes may have affected our results. Second, in the present study, we only investigated latent profiles via self-reports. There is an inherent problem in the use of self-reports as indirect indicators of this phenomenon. In addition, these measures have been associated with stigmatization. Third, here we have considered three specific psychometric indicators (ESQUIZO-Qr, PQ-B, and MDQ) as tentative indices of risk for psychosis. However, it would also be interesting to add other relevant variables as cannabis use, trauma experiences, etc., in order to improve theoretically the predictive capacity of the model. Fourth, the latent structure found here is clearly limited by the tool used and constructs measured. Finally, it should be kept in mind that this study was cross-sectional, and so we cannot make cause-effect inferences.

## Conclusions

Despite these limitations, our study is one of the few investigations to use LPA and multiple psychosis liability indices to identify extended psychosis phenotypes in a representative sample of adolescents. This study allows us to improve the early identification of youths at risk of serious mental disorders in school-based settings in order to reduce the incidence and burden associated with these kinds of mental health problems and help affected young people to succeed academically, socially, behaviorally, and emotionally. Future studies may start to analyse the risk for psychosis beyond the scope of clinical health services such as in school settings, as well as to assess this kind of subclinical experiences with new digital procedures through ambulatory assessment.
